# Prenatal diagnosis of left ventricular aneurysm

**DOI:** 10.4103/0971-3026.45353

**Published:** 2009-02

**Authors:** K. Balakumar

**Affiliations:** Department of Ultrasonography, Balku's Scan, PVS Hospital, Calicut -673 002, Kerala, India

**Keywords:** Congenital cardiac defects, fetal echocardiography, fetal left ventricular aneurysm, prenatal ultrasonography

## Abstract

Fetal cardiac anomalies involving the ventricular and atrial septa, outflow tracts, chambers, and valves are often encountered in routine screening. However, the prenatal detection of a fetal left ventricular aneurysm is rare. This report describes the case of a left ventricular aneurysm that was diagnosed at 24 weeks of gestation; the diagnosis was later confirmed by postnatal echocardiography. This case is reported because of its rarity and the characteristic echocardiographic findings. An early specific antenatal USG diagnosis helps in prognostication and in counseling of the parents.

## Introduction

We would like to report the case of a left ventricular aneurysm diagnosed at 24 weeks of gestation. The diagnosis was eventually confirmed on postnatal echocardiography.

## Case Report

**Figure 1:**
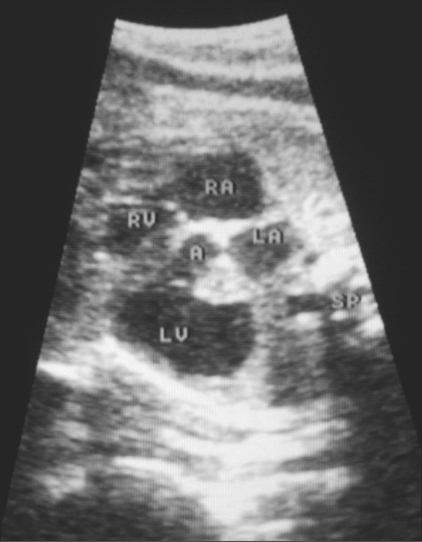
The four-chamber view of the fetal heart shows normal-sized atria (RA - right atrium, LA - left atrium), and right ventricle (RV). The left ventricle (LV) is massively dilated and the aorta (A) can be traced arising from it. The fetal head is in the lower uterine cavity and the fetal spine (SP) is along the left side of the mother

**Figure 2:**
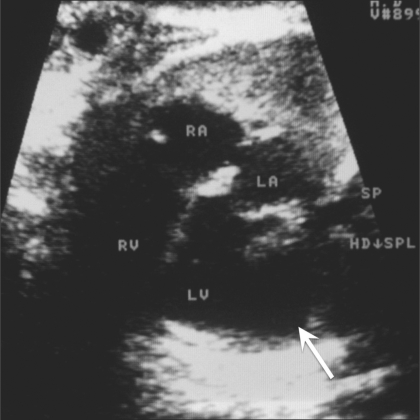
An enlarged representation of the four-chamber view shows the intact interventricular septum between the ventricles (RV - right ventricle, LV - left ventricle). The foramen ovale is seen between the atria (RA - right atrium, LA - left atrium). The aneurysm is directed posteriorly and to the left (arrow); it has a very thin wall and a broad communication with the ventricle. The aneurysmal dimensions remained almost unchanged during ventricular contraction, which was suggestive of dyskinesia. The fetal head (HD) is in the lower pole and the spine (SPL) is along the maternal left side.

**Figure 3:**
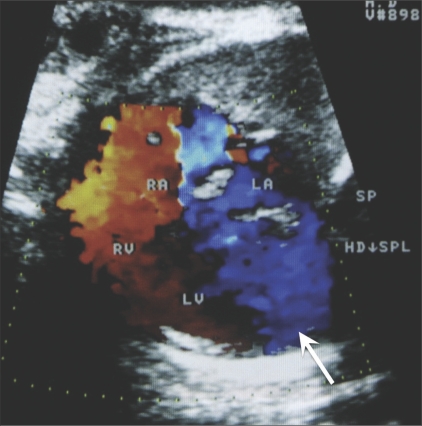
Color Doppler imaging shows massive tricuspid regurgitation and the dyskinetic left ventricle and aneurysm (arrow) (RA - right atrium, RV - right ventricle, LA - left atrium, LV - left ventricle)

An asymptomatic young (26-year-old) primigravida was referred to us at 24 weeks of gestation for a routine antenatal USG. Her personal and family histories were noncontributory. She gave no history of exposure to any teratogen or of a recent infection. The USG showed a normal placenta, normal volume of liquor, and normal biometry corresponding to 24 weeks' gestation. The cardiac four-chamber view was abnormal because of the presence of a dilated left ventricle with a ‘boot-shaped’ appearance [Figure 1]. We carried out a detailed fetal echocardiographic examination. The cardiac situs was normal, with a near-normal axis and moderate cardiomegaly. The disproportionately dilated left ventricle had an abnormal shape because of a pouch-like extension towards the left and posterior aspect. The thin wall of this sac-like projection was continuous with the left ventricular wall [Figure 2]. The dyskinetic left ventricle communicated with the pouch through a wide mouth. Color flow imaging showed abnormal low-velocity turbulent flow in this pouch and no demonstrable contraction during systole [Figure 3]. These features were diagnostic of a left ventricular aneurysm. There was severe mitral and tricuspid incompetence in this fetus. The outflow tracts, septa, ductus arteriosus, and descending aorta were all normal.

## Discussion

With the advent of sophisticated machines and software, fetal echocardiography has become a very useful investigation. The detection rates of cardiac anomalies are increasing, as also the specificity of the diagnoses.[1] In one recent report, the overall prevalence of cardiac anomalies at birth was 7.8/1000, inclusive of still births, live births and termination of pregnancy due to congenital heart disease.[2] Ventricular aneurysm is a recent addition to the list of diseases that can be diagnosed before birth; the first case was reported in 1990[3] and approximately 20 cases have been reported till 2005.[4] The reported incidence is 0.5 per 100,000 live births. Classically, the USG diagnosis of a ventricular aneurysm is made based on the presence of a wide-mouthed ventricular outpouching along with the presence of paradoxical systolic expansion, though this may not always be seen. The aneurysm may arise from the ventricular septum, the wall, or the apical region. This fetus showed no other intra- or extracardiac anomaly; other anomalies are only rarely associated with such aneurysms.[5]

A ventricular diverticulum has to be differentiated from an aneurysm. A diverticulum is usually a small finger-like, synchronously contracting, narrow projection, which shows the same thickness and layering as the rest of the myocardium.[6] Fetuses with an isolated diverticulum have a good prognosis if the associated complications are treated.[7,8] Sometimes they have midline anomalies as in pentalogy of Cantrell. Ebstein's anomaly, cardiomyopathy, outflow tract obstruction, and substantial atrioventricular regurgitation may all be confused with a right ventricular diverticulum.[9] The differential diagnosis of ventricular aneurysm also includes post-ischemic aneurysm. Other lesions that can occur in the vicinity, such as intrathoracic cystic anomalies (e.g., intrathoracic lung cysts), diaphragmatic hernia, absent right sided pericardium, Uhl's anomaly, and right ventricular dysplasia, should also be considered in the differential diagnoses.

The prognosis of a fetal left ventricular aneurysm depends on the time of detection, the size and the progression of the aneurysm, the presence or absence of compression of the fetal lungs, involvement of the mitral opening, reversal of atrial shunt, associated cardiac failure, and the presence of myocardial damage or connective tissue disorders.[5,10,11]

This fetus was delivered by a Cesarian section for purely obstetric indications, and the postnatal detailed echocardiography confirmed the antenatal diagnosis.
